# Comparison of RCI001 and corticosteroid on the effects on intraocular pressure in mice

**DOI:** 10.3389/fmed.2023.1256569

**Published:** 2023-10-09

**Authors:** Soo Hyun Kim, Young-ah Ku, Chungkwon Yoo, Yong Ho Kim, Dong Hyun Kim

**Affiliations:** ^1^Department of Ophthalmology, Korea University College of Medicine, Seoul, Republic of Korea; ^2^RudaCure Co., Ltd., Incheon, Republic of Korea; ^3^Gachon Pain Center and Department of Physiology, Gachon University College of Medicine, Incheon, Republic of Korea

**Keywords:** RCI001, dry eye disease, corticosteroid, intraocular pressure, ocular surface disease

## Abstract

**Purpose:**

RCI001, a novel therapeutic candidate for the treatment of ocular inflammatory diseases, have demonstrated remarkable anti-inflammatory and antioxidant effects in various ocular experimental models. This study was to evaluate the effects of RCI001 on intraocular pressure (IOP) and compare them with those of corticosteroids in experimental mouse models.

**Methods:**

Experimental mice were randomly divided into naïve, phosphate-buffered saline (PBS), 0.1% dexamethasone (DEX-1), and 1% RCI001 (RCI) groups, and each reagent was pipetted into the right eye of the mouse at 10 μL thrice daily for 5 weeks. In addition, 20 μL of 0.1% dexamethasone was injected subconjunctivally into the right eye once weekly for 5 weeks in the DEX-2 group. The IOP was measured under anesthesia at baseline and twice weekly for 5 weeks. The △IOP (%) was defined as the change in IOP from baseline [△IOP (%) = (IOP_*week*5_–IOP_*baseline*_)/IOP_*baseline*_ × 100%]. The anterior segments were clinically and histologically examined.

**Results:**

There was no significant increase in IOP and △IOP (%) [values by week 3 (day 21) in any of the groups]. However, IOP and △IOP (%) in the DEX-2 group tended to increase slightly after day 10 compared with baseline. Compared with baseline IOP values, the DEX-1 group showed a statistically significant increase in IOP at weeks 4 and 5, and the DEX-2 group at week 5. The △IOP (%) of the DEX-1 and DEX-2 groups (%) at week 5 were 38.2% ± 5.8% and 38.4 ± 4.6%, respectively. However, the IOP in the RCI group did not increase significantly until week 5. The RCI group did not show notable corneal changes, such as epithelial defects or stromal opacities, at week 5. In addition, hematoxylin and eosin (H&E) staining of corneas in the RCI group revealed healthy corneal epithelial, stromal, and endothelial integrity.

**Conclusion:**

Long-term use of RCI001 did not induce significant IOP elevation or ocular surface changes, whereas topical corticosteroids significantly increased the IOP. Therefore, RCI001 may be an effective anti-inflammatory agent with a low risk of drug-induced IOP elevation.

## 1. Introduction

The ocular surface, which consists of the cornea, limbus, conjunctiva, and tear film, is in constant contact with various antigens and pathogens from the external environment (1), and an intact ocular surface is essential for healthy ocular function. Ocular surface disease (OSD) occurs when the integrity of the ocular surface is compromised and is known to be directly related to inflammation (2). OSDs include dry eye disease (DED), allergic conjunctivitis, chemical burns, ocular graft-versus-host disease, and Stevens-Johnson syndrome. Increased tear osmolarity, tear film instability, and ocular surface inflammation play important roles in OSD (2, 3). Recently, OSD has become a major societal burden. The annual medical cost per patient with dry eyes in the United States is estimated to be 783 USD, with a total cost of approximately 3.84 USD billion per year (4).

Corticosteroids are among the most commonly used drugs worldwide for the treatment of various inflammatory diseases. Topical corticosteroids are also used to treat various ocular surface inflammatory diseases such as DED, allergic conjunctivitis, and chemical burns (5). However, the long-term use of corticosteroids leads to serious ocular complications, including steroid-induced glaucoma, delayed corneal epithelial healing, and cataract formation. In particular, steroid-induced glaucoma is a major concern for ophthalmologists using corticosteroids because the glaucomatous optic nerve damage caused by steroid-induced glaucoma is irreversible (6, 7).

Thus, RCI001 is a novel drug candidate for the treatment of inflammatory eye diseases. The active ingredient of RCI001, 8-oxo-2′-deoxyguanosine (8-oxo-dG), is an oxidative derivative of deoxyguanosine released from the oxidative damage of DNA chemical reactions (8, 9). Administration of RCI001 has shown remarkable anti-inflammatory and antioxidant effects in various inflammation models (8, 10–13). Our previous studies also showed that the anti-inflammatory effects of RCI001 were comparable to those of 1% prednisolone acetate, one of the potential commercially available anti-inflammatory eye drops (14).

As RCI001 has shown potent anti-inflammatory and antioxidant effects in several experimental models of OSD, it has promising potential as an excellent therapeutic agent and a replacement for topical corticosteroids in OSD. However, no study has investigated the effects of RCI001 on intraocular pressure (IOP). The objective of this study was to evaluate the effects of RCI001 on IOP and compare them with those of corticosteroids in experimental mouse models.

## 2. Materials and methods

### 2.1. Animals and experimental design

All experimental animals were treated in accordance with the National Institutes of Health Guide for the Care and Use of Laboratory animals, and the experimental protocols were approved by the Institutional Animal Care and Use Committee (LCDI-2018-0083). The animals were treated in strict accordance with the Association for Research in Vision and Ophthalmology Statement for the Use of Animals in Ophthalmic and Vision Research.

Thirteen-week-old female C57BL/6 mice (*n* = 45) were obtained from Dae Han Biolink Co., Ltd (Eumseong, Republic of Korea). All mice had free access to water and food. Anesthesia was induced by intraperitoneal injection of sodium pentobarbital (65 mg/kg) (Entobar, Hanlim Pharmaceuticals Co., Seoul, Republic of Korea).

The animals were randomly divided into five groups. The following reagents were administered to the mice: phosphate-buffered saline (PBS), 0.1% dexamethasone (Yuhan Co., Seoul, Republic of Korea, DEX-1), and 1% RCI001 (RCI). Nothing was done in the naïve group. RCI001 was dissolved in normal saline to a purity of > 95%. The naïve group was included as the control group. Each reagent was pipetted into the right eye of the mouse at 10 μL three times daily for 5 weeks. In addition, 20 μL of 0.1% dexamethasone (Yuhan Co., Seoul, Republic of Korea) was injected subconjunctivally into the right eye once weekly for 5 weeks using a 31G insulin syringe (BD, USA) under anesthesia in the DEX-2 group.

### 2.2. IOP measurement

**FIGURE 1:**
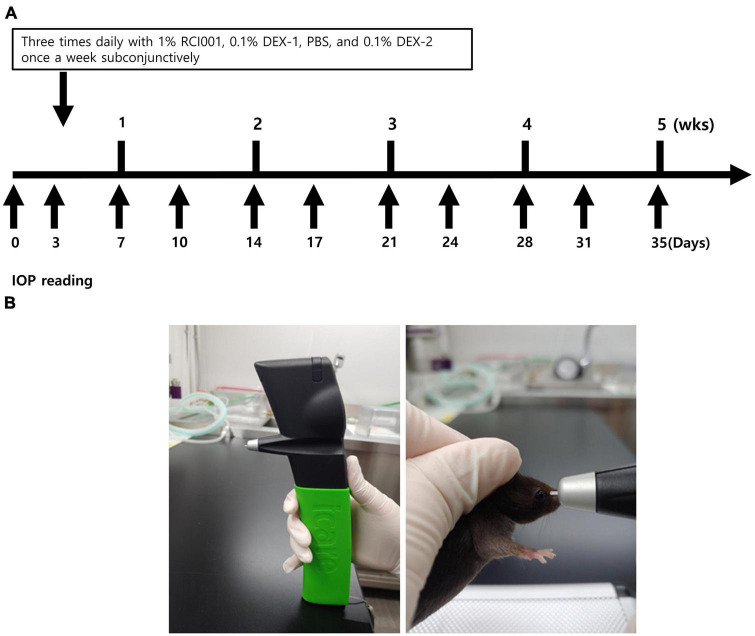
**(A)** Schematic diagram about drug applications and IOP measurements. Eye drops are administered three times daily in naïve, PBS, RCI001, DEX-1 groups daily, and 0.1% dexamethasone is injected into subconjunctiva in DEX-2 group once weekly. IOP is measured twice a week until 35 days. **(B)** Methods of IOP measurement with TonoLab tonometer. The instrument is placed vertically with the surface, and the distance between the probe and eye is 0.5 cm. IOP is measured after 15 s of stabilization to avoid restraint-induced iatrogenic IOP elevation. IOP, intraocular pressure; PBS, phosphate-buffered saline; DEX-1, eye drops containing 0.1% dexamethasone; DEX-2, subconjunctival injection of 0.1% dexamethasone.

The IOP was measured under anesthesia at baseline and twice weekly for 5 weeks (Figure 1A). During the daytime (10 AM to 2 PM), conscious IOP was measured in behaviorally trained mice using a TonoLab rebound tonometer (iCare, Franconia, NH, USA) at baseline and twice weekly for 5 weeks, according to a previously published method (15). The instrument was placed vertically with respect to the surface, and the distance between the probe and eye was 0.5 cm (Figure 1B). IOP was measured after 15 s of stabilization to avoid restraint-induced IOP elevation. The IOP in the right eye was recorded when the same measurement was obtained three times at an interval of 3–5 s. IOP was measured by the same experienced examiner (Y. Ku). Until week 3, the IOP measured twice weekly was analyzed and referred to as short-term IOP change. Long-term IOP change was defined as IOP measurements until week 5, and data measured once weekly were analyzed. IOP change was calculated as:


$$\mathrm{IOP}\mathrm{change}={\mathrm{IOP}}_{\mathrm{week5}}-{\mathrm{IOP}}_{\mathrm{baseline}}$$

The percentage increase was determined according to the following formula:


$$△\mathrm{IOP}\left(\%\right)=\left({\mathrm{IOP}}_{\mathrm{week5}}-{\mathrm{IOP}}_{\mathrm{baseline}}\right)/{\mathrm{IOP}}_{\mathrm{baseline}}\times 100\%$$

IOP elevation was considered to be ≥ 30% from the baseline.

### 2.3. Clinical and histological examinations of anterior segments in RCI001 group

The anterior segments of the mice in the RCI001 group were clinically evaluated by an experienced investigator (Y. Ku) using a handheld slit-lamp biomicroscope (SL-17, Kowa, Tokyo, Japan) at week 5. The ocular surface was photographed using an OPMI LUMERA 300 (Carl Zeiss, Germany) and EzRecorder 130 (AVerMedia, New Taipei City, Taiwan). In addition, these eyeballs were enucleated, fixed in 10% formalin, embedded in paraffin, sectioned at 4 μm thickness, and stained with hematoxylin and eosin (H&E).

### 2.4. Statistical analysis

All IOP values were compared with baseline in each group using Wilcoxon signed rank and Friedman tests (GraphPad Prism^®^ software, Inc., La Jolla, CA, USA) and are shown as mean ± standard error of the mean (SEM). The Mann–Whitney U test was used to compare the DEX-1, DEX-2, and RIC groups. A *p*-value of < 0.05 was considered statistically significant.

## 3. Results

Seven mice were included in the naïve, PBS, RCI001, and DEX-2 groups, respectively, and ten in the DEX-1 group. The baseline IOP values of the naïve, PBS, RCI001, DEX-1, and DEX-2 groups were 10.1 ± 0.1, 10.3 ± 0.2, 10.4 ± 0.2, 10.5 ± 0.2, and 10.6 ± 0.4 mmHg, respectively. There were no significant differences in the baseline IOP between the groups (Kruskal–Wallis test, *p* = 0.658, Table 1).

**TABLE 1:** Distribution of intraocular pressure in naïve, PBS, RCI001, DEX-1, and DEX2 groups for 5 weeks after drug application.

	IOP (mmHg, mean ± SD)					
	**Week 0**	**Week 1**	**Week 2**	**Week 3**	**Week 4**	**Week 5**
Naïve	10.1 ± 0.1	10.0 ± 0.0	10.0 ± 0.0	10.0 ± 0.0	10.1 ± 0.1	10.6 ± 0.3
PBS	10.3 ± 0.2	10.0 ± 0.0	10.1 ± 0.1	10.0 ± 0.0	10.1 ± 0.1	10.9 ± 0.3
RCI001	10.4 ± 0.2	10.0 ± 0.0	10.1 ± 0.1	10.0 ± 0.0	10.9 ± 0.5	10.9 ± 0.3
DEX-1	10.5 ± 0.2	10.4 ± 0.2	10.2 ± 0.2	10.4 ± 0.4	**13.3 ± 0.8** *	**14.5 ± 0.6** **
DEX-2	10.6 ± 0.4	11.1 ± 0.6	11.1 ± 0.6	11.0 ± 0.4	12.1 ± 0.5	**14.6 ± 0.5** *

*P < 0.05; **P < 0.01 (comparison with baseline).

The bold values are significantly elevated values compared with baseline values.

IOP, intraocular pressure; PBS, phosphate-buffered saline; DEX-1, eye drops of 0.1% dexamethasone; DEX-2, subconjunctival injection of 0.1% dexamethasone.

**FIGURE 2:**
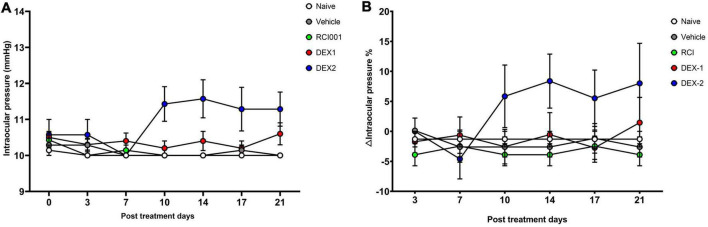
Short-term changes of intraocular pressure in naïve, PBS, RCI001, DEX-1, and DEX2 groups for 5 weeks after drug application. **(A)** IOP values, **(B)** IOP changes percentages from baseline values. IOP, intraocular pressure; PBS, phosphate-buffered saline; DEX-1, eye drops containing 0.1% dexamethasone; DEX-2, subconjunctival injection of 0.1% dexamethasone. Data are presented as means ± standard error (SE).

When intraocular pressure was measured twice weekly until week 3, there was no significant increase in IOP and △IOP (%) values until week 3 (day 21) in all the groups (Figure 2). However, IOP and △IOP (%) in the DEX-2 group tended to increase slightly after day 10 compared with baseline (Figure 2).

**FIGURE 3:**
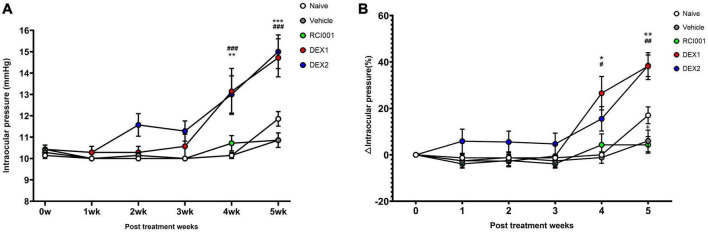
Long-term changes of intraocular pressure in naïve, PBS, RCI001, DEX-1, and DEX2 groups for 5 weeks after drug application. **(A)** IOP values, **(B)** IOP changes percentages from baseline values. IOP, intraocular pressure; PBS, phosphate-buffered saline; DEX-1, eye drops containing 0.1% dexamethasone; DEX-2, subconjunctival injection of 0.1% dexamethasone. Data are presented as means ± standard error (SE). ^#^*p* < 0.05, ^##^*p* < 0.01, and ^###^*p* < 0.005 (comparison between DEX-1 and naïve groups); *p < 0.05, **p < 0.01, and ***p < 0.005 (comparison between DEX-2 and naïve groups).

Compared with baseline IOP values, the DEX-1 group showed a statistically significant increase in IOP at weeks 4 and 5, and the DEX-2 group at week 5. However, the IOP in the RCI001 group did not significantly increase until week 5. At week 5, the IOP of naïve, PBS, RCI001, DEX-1, and DEX-2 groups were 11.9 ± 0.3, 10.9 ± 0.3, 10.9 ± 0.3, 14.5 ± 0.6, and 14.6 ± 0.5 mmHg, respectively (Table 1). The IOP of DEX-1 and DEX-2 groups at week 5 increased significantly by 4.0 ± 0.6 and 4.0 ± 0.4 mmHg, respectively, compared with their baseline IOPs [*p* = 0.002/0.016 (DEX-1/DEX-2), Wilcoxon signed-rank test] (Table 2). There were no significant IOP changes in the RCI001 group at week 5 compared with their baseline values (*p* = 0.257). In addition, the IOPs of the DEX-1 and DEX-2 groups increased significantly compared with the naive group at weeks 4 and 5 [*p* < 0.001/< 0.01 (DEX-1/DEX-2) at week 4 and *p* < 0.0001 (DEX-1/DEX-2) at week 5, Figure 3A]. The △IOP (%) of the DEX-1 and DEX-2 groups was also higher than that of the naïve group at weeks 4 and 5 [*p* < 0.05 (DEX-1/DEX-2) at week 4 and *p* < 0.0001 (DEX-1/DEX-2) at week 5, Figure 3B].

**TABLE 2:** Changes of intraocular pressure values and percentages in naïve, PBS, RCI001, DEX-1, and DEX2 groups for 5 weeks after drug application.

	IOP at week 5 (mmHg)	▲ IOP (mmHg) (IOP_*week* 5_–IOP_*week* 0_)	▲ IOP percentage (%) (IOP_*week* 5_–IOP_*week* 0_/IOP_*week* 0_)	*P*-value† (▲ IOP)	*P*-value‡ (comparison with RCI001)
Naive	10.6 ± 0.3	0.4 ± 0.4	4.4 ± 3.6	0.500	0.367
PBS	10.9 ± 0.3	0.6 ± 0.5	6.0 ± 4.7	0.344	0.500
RCI001	10.9 ± 0.3	0.4 ± 0.4	4.3 ± 3.6	0.257	NA
DEX-1	**14.5 ± 0.6**	**4.0 ± 0.6**	**38.2 ± 5.8**	**0.002**	**< 0.001**
DEX-2	**14.6 ± 0.5**	**4.0 ± 0.4**	**38.4 ± 4.6**	**0.016**	**< 0.001**

^†^Comparison with baseline IOP.

^‡^Comparison with RCI001 at week 5.

The bold values are significantly elevated values compared with baseline values.

IOP, intraocular pressure; PBS, phosphate-buffered saline; DEX-1, eye drops of 0.1% dexamethasone; DEX-2, subconjunctival injection of 0.1% dexamethasone.

Table 2 shows the detailed changes in IOP and △IOP (%) 5 weeks after drug application. The △IOP (%) of the DEX-1 and DEX-2 groups were 38.2 ± 5.8% and 38.4 ± 4.6%, respectively, at week 5. Compared with the IOPs of the RCI001 group, those of the DEX-1 and DEX-2 groups also increased significantly (*p* < 0.001, Table 2). The IOPs of the naïve and PBS groups were not significantly different from those of the RCI001 group (*p* = 0.367 and 0.500, respectively).

**FIGURE 4:**
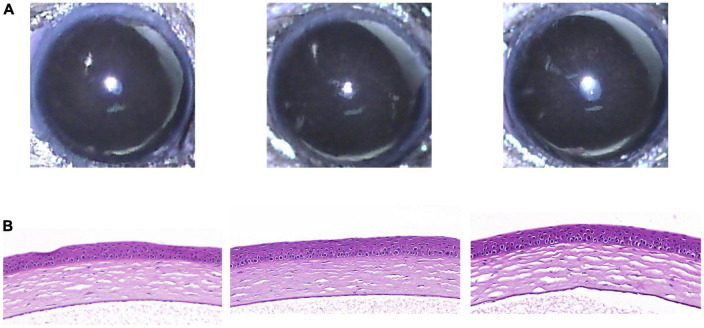
Clinical and histological images of cornea in RCI001 group at week 5. **(A)** Anterior segment photographs of RCI001 group at week 5. There are no abnormal changes observed, such as corneal edema or opacity. **(B)** H&E staining of RCI001 group at week 5 shows healthy corneal epithelial findings and intact stromal and endothelial integrity. H&E, hematoxylin and eosin.

Five weeks after eye drop application, the RCI group showed no significant abnormal changes on the ocular surface, such as corneal epithelial defects or stromal opacity (Figure 4A). In addition, H&E staining of the cornea revealed healthy corneal epithelial, stromal, and endothelial integrity (Figure 4B).

## 4. Discussion

This study demonstrated significant IOP elevations in the DEX-1 and DEX-2 groups (38.2 ± 5.8 and 38.4 ± 4.6%, respectively) compared with baseline, whereas the RCI group showed no significant change in IOP until week 5. In addition, long-term application of RCI001 for 5 weeks did not induce notable adverse changes on the ocular surface.

Corticosteroids initiate anti-inflammatory effects by binding to cytoplasmic glucocorticoid receptors (GR). The corticosteroid-GR complex translocates to the nucleus and modifies the genetic phenotype of the protein, thereby increasing the production of anti-inflammatory substances and suppressing the action of pro-inflammatory proteins (16). It also acts rapidly on inflammatory cells and promotes cell adhesion via non-genomic chemical mechanisms (17). This results in the inhibition of cellular infiltration, microvascular dilation, fibroblast production, collagen accumulation, and scar formation. In the anterior segment, it is used to treat various ocular surface inflammatory diseases, such as inflammation caused by surgery, trauma, dry eyes, conjunctivitis, uveitis, transplant rejection, scleritis, and episcleritis (18–20). It is also widely used in various posterior segment diseases such as macular edema after vascular occlusion, diabetic macular edema, and macular degeneration (21, 22). In contrast, RCI001 exerted anti-inflammatory effects by inhibiting the Rac1 and NLRP inflammasome/IL-1b axes (9). Accordingly, RCI001 may also suppress the activation of innate immune cells, such as neutrophils and macrophages, and exhibit potent anti-inflammatory effects similar to those of corticosteroids (23).

Steroid-induced IOP elevation was first reported in a previous study in 1950 showing IOP elevation with systemic adrenocorticotrophic hormones (24), followed by a case report describing IOP elevation with topical cortisone administration (25). Steroid-induced IOP elevation can be attributed to the microstructure of the trabecular meshwork (TM), decreased protease function, TM endothelial phagocytosis, and increased resistance to aqueous flow as substances accumulate in the TM (25). The degree of IOP elevation depends on the potency, dose, and route of glucocorticoid administration (26, 27). In general, IOP elevation greater than 10 mmHg from baseline is considered steroid-induced ocular hypertension, and those with such elevation are called “steroid responders.” Steroid-induced IOP elevation occurs in both adults and children. A previous study reported that 25% of children with acquired glaucoma had steroid-induced glaucoma (28). As the IOP increases with long-term steroid use, the risk of optic nerve damage increases, leading to irreversible glaucomatous optic neuropathy (29). In a previous study, 2.8% of eyes with corticosteroid-induced IOP elevation developed glaucoma (30). A recent study by Razali et al. (31) showed that 0.1% topical dexamethasone application twice daily until week 9 increased IOP by 36–43% from week 4 to week 9. Zode et al. (32) also administered 0.1% topical dexamethasone eye drops thrice daily to C57BL/6 mice and demonstrated an elevated IOP of 3.3 mmHg at week 2 and a continuous increase to 7.7 mmHg at week 6. The absolute increase in IOP when periocular conjunctival fornix injections of dexamethasone 21-acetate were administered to C57BL/6J mice was approximately 4 mmHg for 4 weeks (6). Our present study showed a 38.2 and 38.4% increase in IOP at week 5 compared with baseline with 0.1% dexamethasone eye drops and subconjunctival injections, respectively. These results were consistent with those of previous studies.

This study had several limitations. First, the sample size was small. Second, the IOP was not assessed after 5 weeks. Third, the dose-dependent adverse effects of RCI001 on IOP were not evaluated. Fourth, the cataract formation, another ocular side effect of corticosteroids, was not evaluated. Nevertheless, this study showed that RCI001, which has anti-inflammatory properties comparable to those of corticosteroids, may not cause an increase in IOP even with long-term use.

In conclusion, the long-term use of topical corticosteroids significantly increased IOP, whereas RCI001 did not induce significant IOP elevation or ocular surface changes. Therefore, we believe that RCI001 may be a promising candidate for an effective anti-inflammatory agent with fewer long-term complications than topical corticosteroids.

## Data availability statement

The original contributions presented in this study are included in the article/supplementary material, further inquiries can be directed to the corresponding authors.

## Ethics statement

The animal studies were approved by Institutional Animal Care and Use Committee (LCDI-2018-0083). The studies were conducted in accordance with the local legislation and institutional requirements. Written informed consent was obtained from the owners for the participation of their animals in this study.

## Author contributions

SK: Formal analysis, Investigation, Validation, Writing – original draft. Y-aK: Data curation, Investigation, Methodology, Visualization, Writing – review and editing. CY: Supervision, Writing – review and editing. YK: Conceptualization, Data curation, Funding acquisition, Methodology, Project administration, Resource, Supervision, Writing – review and editing. DK: Conceptualization, Formal analysis, Funding acquisition, Investigation, Methodology, Project administration, Resources, Supervision, Writing – review and editing.
